# Health literacy and household financial loss on malaria treatment for children under five in Ghana: a patients’ perspective

**DOI:** 10.1093/inthealth/ihae022

**Published:** 2024-04-02

**Authors:** Millicent Ofori Boateng, Derek Asuman, Nuworza Kugbey, Padmore Adusei Amoah, Peter Agyei-Baffour, Ulrika Enemark

**Affiliations:** Faculty of Health, Department of Public Health, Aarhus University, Bartholins Alle 2, Building 1260, DK 8000 Aarhus C, Aarhus, Denmark; School of Public Health, Department of Community Health, Ensign Global College, P.O. Box AK 136, Akosombo, Eastern Region, Ghana; Health Economics Unit, Lund University, Medicon Village 301:5, Scheelevagen 2, 223 81, Box 117, 221 00 Lund, Sweden; School of Public Health, Department of Community Health, Ensign Global College, P.O. Box AK 136, Akosombo, Eastern Region, Ghana; Department of General Studies, School of Natural and Environmental Sciences, University of Environment and Sustainable Development, PMB, Somanya, Ghana; Department of Psychology, Institute of Policy Studies, School of Graduate Studies, Lingnan University, 8 Castle Peak Road, Lingnan, Tuen Mun, Hong Kong; Department of Occupational Health, School of Public Health, Kwame Nkrumah University of Science and Technology, Private Mail Bage, University Post Office, Kumasi, Ghana; Faculty of Health, Department of Public Health, Aarhus University, Bartholins Alle 2, Building 1260, DK 8000 Aarhus C, Aarhus, Denmark

**Keywords:** caregivers, children, health literacy, household cost, malaria

## Abstract

**Background:**

Inadequate health literacy increases medical costs and leads to poor health outcomes. However, there is a paucity of empirical evidence of such associations in sub-Saharan Africa. This study investigates how the household cost of malaria in children under five in Ghana varies based on different levels of health literacy.

**Methods:**

A cross-sectional survey involving 1270 caregivers of children under five was conducted. The survey included health literacy questionnaire and several pieces of sociodemographic and behavioural variables.

**Results:**

We created seven caregiver health literacy profiles by scoring nine dimensions. The mean total cost for managing malaria among respondents was US$20.29 per episode. The total household cost for caregivers with high health literacy (Profile 1) (US$24.77) was higher than all other profiles, with the lowest cost (US$17.93) among the low health literacy profile (Profile 6). Compared with Profile 4, caregivers with high health literacy (Profile 1) spent more on managing malaria in children, while those with the lowest health literacy (Profile 7) spent less.

**Conclusion:**

The current study presents a snapshot of malaria treatment costs, and argues that low health literacy may lead to increased costs due to possible reinfections from delayed healthcare use. There is a need for longitudinal studies to understand causal relationship between health literacy and household expenses on malaria treatment to inform policy development and interventions.

**Lay Summary:**

This study explores the impact of caregiver health literacy levels on the cost of managing malaria incidents in children under five in Ghana. High health-literate caregivers incurred the highest total household cost at US$24.77, with US$17.93 incurred by lower health-literate caregivers per malaria episode.

## Introduction

Health literacy has recently gained attention in health promotion and health services provision, enabling individuals and groups with differences to manage their health effectively.^1^ Health literacy is a multifaceted concept with diverse perspectives regarding its definition.^2^ Generally, it encompasses the cognitive and social skills that determine individuals' motivation and ability to gain access to, understand and apply information in ways that promote and maintain good health.^3^ According to Sørensen et al., maintaining good health includes making health decisions concerning healthcare, disease prevention and health promotion.^2^ The WHO defines health literacy as people's ability to acquire personal knowledge and skills from everyday activities and interactions spanning multiple generations.^4^ Organisational structures and resource availability are crucial in shaping personal knowledge and competencies, enabling individuals to access, comprehend, assess and apply information and services for their health and well-being.^4^ For instance, as a social determinant of health, health literacy was described as a social vaccine against infection and infodemics during the COVID-19 pandemic.^5^ The intricacies inherent in this concept have resulted in the development of various definitions, dimensions and measurement tools specifically tailored to address its contextual and content-specific nature.^1,2,6,7^ These tools encompass multidimensional questionnaires, such as the Health Literacy Survey-European Union, which covers critical facets of the concept, including access, understanding, appraisal and application of information.^2,8^ Another widely used tool is the Health Literacy Questionnaire (HLQ), which assesses nine dimensions of health literacy.^7^ Both questionnaires are employed globally in assessing health literacy.^9–11^

Low health literacy can significantly impact caregivers' ability to understand important information, develop positive attitudes and show appropriate behaviour, such as following medical advice.^12^ This can, in turn, affect the health outcomes of their children in various areas, including disease prevention, acute illness management and chronic illness care.^1,12,13^ To reduce the negative impact of low health literacy, interventions are designed and implemented to improve the health literacy of caregivers to enhance the health status of their children.^12,14^

Inadequate health literacy is associated with high needs for disease management and frequent use of medical services among the old and those belonging to a minority group (e.g. an ethnic minority).^15–18^ Thus, inadequate health literacy will probably result in higher medical costs and poor health outcomes.^19^ Haun et al., estimated that the three-year cost associated with medical and pharmacy expenses was US$143 million dollars more for veterans with marginal and inadequate health literacy than those with adequate health literacy.^20^ Likewise, a population-based study in Ghana showed that high levels of health literacy enabled householders to reduce out-of-pocket health expenditures by enhancing their access to health insurance.^21^ However, there is limited knowledge of the impact of health literacy on household healthcare costs, especially in malaria-endemic countries, indicating the need for further research.

Over the years, there has been a global reduction in the malaria burden, yet malaria remains a global public health problem.^22^ In Ghana, malaria is one of the leading causes of morbidity and mortality. In 2018, malaria accounted for 4% of the disease burden in Ghana and 7% of the malaria disease burden in West Africa.^23^ Malaria is not close to eradication in Ghana. However, there has been a fall in the proportion of under-five deaths due to malaria from 15% in 2010 to 13% in 2018.^23^ The economic burden of malaria can be catastrophic costs to households. For instance, a study in Ghana showed that about 5% of respondents spent >5% of their annual income on the treatment of malaria, which was described as a catastrophic cost.^24^ Another study by Tawiah et al. reported that the average cost to households was US$14.61 per patient per malaria episode in Ghana.^25^ The introduction of the National Health Insurance Scheme (NHIS) in 2003 was expected to alleviate the economic burden of malaria, especially for poorer households. However, factors such as proximity to the health facilities reduce the functionality of this insurance coverage. Fenny et al. reported that about 15% of insured patients chose to use informal healthcare over formal healthcare due to the distance to the nearest health facility.^26^ In addition, unfamiliarity with insurance validity and renewal dates also contributed to the low active enrolment of the Ghana NHIS.^27^ Despite the vast knowledge of malaria and its costs, there needs to be more research on how health literacy levels influence costs in the treatment of malaria in sub-Saharan Africa. This study seeks to examine: (i) the health literacy profiles of the caregivers of children aged <5 years with malaria; (ii) the household cost associated with malaria; (iii) sociodemographic characteristics associated with the household cost; and (iv) the association between caregivers' health literacy and household cost after controlling for other covariates.

## Methods

### Study design

This is a cross-sectional study, and the data were collected during November and December 2017. We collected data from caregivers with children aged <5 y.

### Sample

The study adopted a multi-stage method to select caregivers of children under five. In the first stage, we purposefully selected two subdistricts (highly populated and less populated) from the two study districts (Ejisu-Juaben and Kwabre East) in the Ashanti Region. Second, we randomly selected nine subdistricts with representations from urban and rural subdistricts. During the third stage, interviewers were allocated to the selected communities, and at the entrance of each community, every other household in the study was visited. The study only included households with children under five. If a house had multiple households under five, one was randomly selected. If a sampled household had >2 caregivers with under-five children, only one was interviewed. Eight research assistants were trained for data collection, and the sample size was equally distributed among them.

Data were collected from 1234 respondents out of a sample size of 1270. Thirty-six caregivers' responses were lost during data synchronisation. This study is part of a larger project that examined the impact of health literacy interventions. The sample size was calculated based on the number of women of reproductive age in two districts, with a 51% improvement in malaria management compared with earlier studies.^28^

### Data collection

We used two questionnaires, the HLQ and a malaria questionnaire, both administered in the local language.^29^ The HLQ was chosen for its excellent psychometric properties, successful use in low-income settings and well-defined translation procedure.^7^ The questionnaire also includes dimensions relevant to the study site's social context, such as ‘social support for health’.^3,7^

The malaria questionnaire included questions on recent malaria episodes in children under five. Caregivers were asked about the sequence of pathways of seeking healthcare for the child (treatment pathways) during an episode of malaria and the costs of treatments, including transport, consultations and medications. Other questions covered the sociodemographic characteristics of the respondents. From the patient's perspective, the study analysed household treatment costs for children under five who had malaria in the past 6 months. This is because the patient perspective is often used as a broad term, not limited to the individual patient, but instead includes a household perspective, which resonates with this study's methods.

### Study variables

#### Outcome variable

Treatment costs included direct and indirect costs incurred about the chosen treatment pathway for the latest malaria episode, which occurred within the past 6 months. Thus, direct costs cover consultation fees paid, transport and medical costs for both formal and informal care. Indirect costs are measured as the value of days lost to care using self-reported daily income. As measured in this study, the treatment costs represent the financial loss rather than the health expenditures. Although we collected cost data in Ghana Cedis, these are presented in this study using US dollars based on the 2017 currency conversion rate (GH1.00=US$0.22).

#### Explanatory variable

Health literacy is presented as a continuous variable (average mean score) in nine health literacy scales according to the HLQ.^30^ The nine distinct areas (scales) are:

1. Feeling understood and supported by healthcare providers.
2. Having sufficient information to manage health.
3. Actively managing my health.
4. Social support for health.
5. Appraisal of health information.
6. Ability to actively engage with healthcare providers.
7. Navigating the healthcare system.
8. Ability to find good health information.
9. Understand health information well enough to know what to do.

Each scale has four to six items, totalling 44 items. The nine scales are divided into two parts. The first part (scales 1–5) uses a four-point Likert scale (strongly disagree, disagree, agree, strongly agree), while the second part (scales 6–9) rates the ability to perform various tasks on a five-point Likert scale (cannot do, very difficult, difficult, easy, very easy).

#### Covariates

Covariates were informed by Paasche Orlow and Wolf's causal model on health literacy. The gender of the respondent, years of schooling, valid NHIS membership, the desirability of the use of healthcare and age of the child were adjusted for in the analysis. We defined the desirability of healthcare in two health-seeking pathways: desirable (using formal healthcare as at least a second treatment choice) and undesirable (e.g. self-treatment). ‘Formal healthcare’ includes public and private health facilities. The desirable pathway is based on the Ministry of Health malaria treatment guidelines.^31^

### Data analysis

Data were analysed using STATA version 15 (StataCorp, College Station, Texas, USA). Respondents' demographic characteristics in using healthcare and the mean treatment costs are presented using descriptive statistics. Using cluster analysis, caregivers were grouped into health literacy profiles based on their health literacy scores across the nine scales. The cluster analysis followed Ward's linkage method using the Squared Euclidean distance measure. Because health literacy is measured in nine dimensions using either a four- or five-point Likert scale in this study, the clusters were generated from the Z-score of each of the nine dimensions to neutralise the differences in range scores. Generalised linear model procedures with a log link function and Gamma distribution measured the association of treatment cost and health literacy. This is because treatment cost (dependent variable) is not normally distributed. We ran bivariate analysis for both associations and ran three adjustments: (i) adjusting for all health literacy scales; (ii) adjusting for covariates for each health literacy scale; and (iii) adjusting for all covariates with all nine scales in the model. p<0.05 was used as the threshold for statistical significance.

## Results

### Caregivers' health literacy profiles

The hierarchical cluster analysis produced seven distinct clusters. After discussions on the analysis between two authors, all the clusters were kept because each covered a good number of respondents for the analysis. Table 1 describes the average mean scores of the nine health literacy scales across the seven health literacy profiles.

**Table 1.** Average scores of nine health literacy scales among health literacy profiles

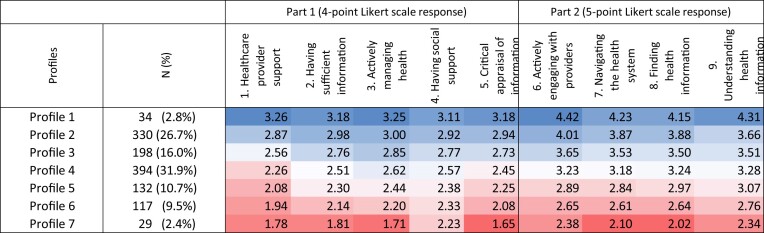

Note: Health literacy scale scores are colour coded from highest (blue) to lowest (red) within each part of the HLQ.

Caregivers within Profile 1 (3%) scored high across all nine scales, and Profile 7 (2%) scored relatively low. Profiles 2 to 6 have moderate to high or moderate to low scores. Most respondents (32%) fell within profile 4 with moderate scores across scales but were better at actively managing their health. Profile 5 had moderate to low scores, with the lowest average score in health provider support. Profiles 4, 5, 6 and 7 had significant challenges with health provider support.

### Sociodemographic profiles of the study participants

Table 2 shows the results of the descriptive statistics of all the demographic characteristics, caregivers' health literacy profiles and the association between all the demographic characteristics and total household costs. Findings showed that most respondents were female (97%), with the majority (73%) of participants aged 25–44 y. Most participants (75%) had <9 y of formal education, and the majority (60%) were employed. Finally, the majority use a desirable pathway (58%), and the majority (82%) have NHIS.

**Table 2.** Mean household costs (US$) and their association with respondent characteristics and health literacy profiles

		Direct cost	Indirect cost	Total cost	
Variable	N (%)	Mean (SD)	Mean (SD)	Mean (SD)	p
Total sample	588(100%)	9.54 (10.12)	11.10 (17.42)	20.29(20.63)	
District					
Ejisu-Juaben	312 (53%)	9.20 (9.99)	10.44 (16.47)	19.41 (19.37)	0.137
Kwabre East	276 (47%)	9.95(10.27)	11.84 (18.44)	21.33 (22.02)	
Gender					
Female	570 (97%)	9.43 (9.98)	11.18 (17.54)	20.26 (20.72)	0.426
Male	18 (3%)	13.43 (14.16)	8.36 (13.23)	21.27 (17.59)	
Age					
15–24 y	126 (21%)	9.93 (10.48)	12.59 (22.42)	21.65 (26.35)	0.683
25–44 y	443 (73%)	9.29 (9.85)	10.74 (15.96)	19.80 (18.79)	
45–69 y	16 (6%)	10.94 (14,29)	9.44 (12.44)	21.17 (21.15)	
Education					
≤9 y	441 (75%)	10.08 (10.29)	11.14 (18.25)	20.92 (21.48)	0.408
>9–≤12 y	134 (23%)	7.72 (9.37)	10.68 (14.39)	18.13 (17.59)	
>12 y	13 (2%)	9.66 (10.10)	14.13 (17.93)	21.16 (19.69)	
Employment					
Employed	355 (60%)	9.23 (10.00)	9.67 (14.54)	18.96 (19.05)	0.026*
Unemployed	233 (40%)	10.03 (10.21)	13.27 (20.92)	22.43 (22.83)	
Long-term illness					
Yes	59 (10%)	9.57 (9.52)	8.48 (12.95)	18.30 (15.58)	0.222
No	529 (90%)	9.53 (10.19)	11.39 (17.84)	20.51 (21.12)	
Language					
English	53 (9%)	10.65 (9.33)	11.29 (16.22)	22.43 (19.73)	0.221
Local	535 (91%)	9.43 (10.19)	11.08 (17.55)	20.08 (20.72)	
Child's age (mo)					
0–12	95 (16%)	8.91 (10.73)	12.07 (16.62)	20.20 (20.69)	0.557
13–24	143 (24%)	9.63 (9.83)	11.66 (17.02)	21.18 (19.81)	
25–36	121 (21%)	10.30 (10.64)	11.00 (17.58)	21.37 (22.42)	
37–48	99 (17%)	9.43 (9.64)	11.27 (20.26)	20.70 (23.23)	
49–60	85 (14%)	10.00 (10.96)	11.02 (18.74)	19.93 (19.76)	
61–71	31 (8%)	7.08 (6.74)	6.40 (7.74)	13.49 (9.61)	
Use of healthcare					
Desirable	343 (58%)	11.70 (11.04)	10.48 (17.32)	22.76 (21.33)	<0.01*
Undesirable	245 (42%)	6.62 (7.84)	11.54 (17.51)	16.96 (19.18)	
NHIS membership					
Yes	481 (82%)	9.68 (9.89)	10.46 (16.66)	19.66 (19.80)	0.069
No	107 (18%)	8.94 (11.05)	13.98 (20.34)	22.96 (23.76)	
Health literacy profiles					
Profile 1	17 (3%)	11.04 (10.50)	12.53 (18.43)	24.77 (21.63)	0.843
Profile 2	165 (28%)	10.26 (9.73)	9.78 (12.70)	20.10 (16.87)	
Profile 3	87 (15%)	10.17 (10.89)	9.77 (13.99)	19.27 (19.16)	
Profile 4	186 (32%)	9.78 (10.48)	12.76 (19.45)	21.74 (22.62)	
Profile 5	58 (10%)	6.77 (9.56)	12.89 (26.59)	19.37 (28.26)	
Profile 6	59 (10%)	8,36 (9,90)	9,66 (15,52)	17,93 (17,55)	
Profile 7	16 (2%)	8,97 (7,42)	9,85 (16,03)	18,81 (18,20)	

Note: Direct cost covers consultation and medical costs, while indirect cost includes the value for days lost to care.

*Statistically significant difference between total cost incurred by users of desirable healthcare and undesirable healthcare users.

### Association between sociodemographic characteristics and total household cost

The average household cost associated with malaria treatment was US$20.29, with an average direct cost of US$9.54 and an indirect cost of US$11.10. Table 2 shows the mean total household cost for malaria in children under five by their caregivers' sociodemographic characteristics and health literacy profiles. Total household costs were higher among older caregivers, caregivers with >12 y of schooling, unemployed caregivers, caregivers without long-term illness, caregivers who spoke English at home, caregivers with children within the 25–36 mo age group, caregivers who chose a desirable treatment pathway and caregivers without national health insurance. Concerning the health literacy profiles, the total household cost for caregivers in Profile 1 (US$24.77) was higher than all other profiles, with the lowest cost in Profile 6 (US$17.93). The mean difference in the total household costs for all the groups was not statistically significant, except for employment and healthcare use (desirable and undesirable).

### Association between health literacy profiles and total household cost using the generalised linear model

After adjusting for all covariates, caregivers from all profiles incurred less household cost on malaria than Profile 4, except for Profile 1 (US$0.24). Caregivers in Profile 6 incurred the least cost (US$0.30), then Profile 7 (US$0.14), compared with Profile 4. Profile 3 incurred less (US$0.11) total household cost on malaria in children than Profile 4. The results are summarised in Table 3.

**Table 3.** Relationship between health literacy profiles of caregivers and total household cost of malaria in children under five

	Unadjusted analysis		Adjusted analysis (sociodemographic covariates)		Adjusted analysis (all covariates)	
Health literacy profiles	Coeff.	95% CI	Coeff.	95% CI	Coeff.	95% CI
Profile 1	0.13	−0.41, 0.67	0.25	−0.31, 0.81	0.24	−0.32, 0.80
Profile 2	−0.08	−0.30, 0.14	−0.02	−0.25, 0.20	−0.01	−0.23, 0.21
Profile 3	−0.12	−0.39, 0.15	−0.10	−0.37, 0.17	−0.11	−0.38, 0.16
Profile 4	Ref.	Ref.	Ref.	Ref.	Ref.	Ref.
Profile 5	−0.12	−0.42, 0.19	−0.13	−0.44, 0.18	−0.11	−0.42, 0.20
Profile 6	−0.19	−0.50, 0.11	−0.26	−0.57, 0.05	−0.30	−0.61, 0.01
Profile 7	−0.14	−0.67, 0.38	−0.21	−0.73, 0.32	−0.14	−0.67, 0.39

Note: Sociodemographic covariates include age of the child, gender of respondent, years of schooling, employment status and reported income. All covariates include reported NHIS subscription and desirable use of healthcare in addition to the sociodemographic covariates.

## Discussion

In the current study, average household costs per malaria episode amounted to US$20.29, of which US$9.54 was in direct treatment costs and US$11.10 was due to lost income. This is slightly above estimates in other studies that measured malaria costs in children under five. Using a model-based analysis, Sicuri et al.,^33^ report an expected average direct treatment cost per case of US$7.85 in 2013, and two studies report direct and indirect costs in 2011 of around US$14.00 (US$13.90, US$14.6).^25,32^ In comparison with costs reported from other studies in Ghana, the differences in costs estimates could be attributable to the differences in study population, increasing costs over the years, inclusion of expenditures for informal care in our study, valuation of indirect costs and differences in recall period.^25,33^ Out of the identified studies on cost estimates for malaria, some focus on estimates for malaria in children, while others report on cost estimates for the general population.^24,25,32–34^ In terms of differences in years of cost estimates, most studies were based on almost 10-y-old data.^32,33^

Comparison of cost estimates across studies is also challenged by different cost components included in the studies; for example, one study reports on only direct costs, others report on both direct and indirect costs; some studies report on both informal and formal treatment costs, and others report on only formal treatment costs.^24,25,32,33,35^ However, where possible, we compared relevant subresults from the studies. Generally, compared with the other cost studies with 2-wk and 1-mo recall periods, our study had a more extended recall period of 6 mo, and this could have influenced the cost reported by respondents.^24,25^ The cost level apart, our study reports the indirect cost as a more significant share of the total costs, which is in line with other studies that report 71%, 80% and 74% of costs as indirect expenditure in the management of malaria.^25,32,36^

Concerning associations between health literacy and the cost of healthcare, the direction did not follow previously reported associations between health literacy and the cost of healthcare.^19–21^ We found that caregivers with higher health literacy incur higher costs per malaria episode than those with lower health literacy. This may be due to their willingness to pay for appropriate care, their ability to pay or a difference in the type of healthcare used between profiles.

### Implications for health service delivery, research and education

The findings from this study have implications for health service delivery, research and education. For healthcare service delivery, improving the health literacy of caregivers of children under five is crucial to their desirable healthcare utilisation, despite the relatively higher associated cost. Improved health literacy was found to be predictive of increased healthcare spending. Health providers should address clients' health literacy needs to improve treatment choices and outcomes, despite potentially higher costs associated with adequate management of malaria among children under five. The findings imply that there is a need to widen the scope of health literacy research by employing a mixed methods approach to understand the magnitude of the influence of health literacy on health outcomes and explain the mechanisms underlying health literacy and health outcomes nexus within the Ghanaian sociocultural context. For education, both clinical and public health education programmes should integrate health literacy into their curriculum. Training and awareness by healthcare providers will likely ensure the acquisition of skills necessary to assess clients' health literacy and inform interventions to address limited health literacy.

### Limitations

The cross-sectional design of our study has limitations. It does not allow for close monitoring of costs from the onset of malaria until the child recovers, and it also does not permit firm conclusions on causal inference. We used a 6-mo recall period to estimate the costs incurred by caregivers, but this is susceptible to recall bias.^37^ Additionally, while we have no reason to believe that inaccuracy would differ by health literacy level, malaria may not have been the best health condition to assess the overall relationship between health literacy and costs. A more complex health issue may have shown different associations.

### Conclusion

The current study suggests that caregivers with high health literacy profiles have higher treatment costs than those with low health literacy. Additionally, appropriate healthcare use leads to higher costs. This study focuses on a snapshot of the costs associated with malaria treatment, but low health literacy may lead to increased costs due to possible reinfections from improper healthcare use. Therefore, a longitudinal study is needed to assess the relationship between health literacy and the costs of managing malaria in children under five in Ghana.

## Data Availability

The datasets used and analysed during the current study are available from the corresponding author upon reasonable request.
